# IGF2BP2‐induced circRNF20 facilitates breast cancer cell proliferation via the HuR/CDCA4 axis

**DOI:** 10.1002/kjm2.12949

**Published:** 2025-02-19

**Authors:** Shu‐Tao Wu, Xiao‐Li Hou, Fei Wang, Wei Sun, Jia‐Jie Chen, Ya‐Sen Cao, Hong Cheng

**Affiliations:** ^1^ Yangzhou University Medical College Yangzhou China; ^2^ Department of Medical Science Yangzhou Polytechnic College Yangzhou China; ^3^ Department of Clinical Laboratory Affiliated Hospital of Yangzhou University Yangzhou China; ^4^ Yangzhou University Medical College, Jiangsu Key Laboratory of Experimental & Translational Non‐coding RNA Research, Institute of Translational Medicine, Yangzhou University Yangzhou China

**Keywords:** breast cancer, CDCA4, circRNF20, HuR, IGF2BP2

## Abstract

This study aimed to explore the mechanism of insulin‐like growth factor 2 mRNA‐binding protein 2 (IGF2BP2) affecting the proliferation of breast cancer (BC) cells. The expression of IGF2BP2, circRNA ring finger protein 20 (circRNF20), and cell division cycle‐associated protein 4 (CDCA4) in human BC cells and normal breast epithelial cells was detected via RT‐qPCR or Western blotting. After IGF2BP2 expression was altered, CCK‐8 assay, colony formation assay, and EdU staining were performed to evaluate changes in the proliferation of BC cells. RNA immunoprecipitation (RIP) assay was used to analyze the binding of circRNF20 to IGF2BP2 or HuR, as well as the binding of HuR to CDCA4. RNA pull‐down confirmed the interaction between circRNF20 and HuR. The stability of circRNF20 was tested after treatment with actinomycin D. A nude mouse xenograft tumor model was established to validate the effect of IGF2BP2 in vivo. IGF2BP2, circRNF20, and CDCA4 were highly expressed in BC cells. Silencing IGF2BP2 decreased the proliferation ability of BC cells. Mechanistically, the binding of IGF2BP2 to circRNF20 prevented circRNF20 degradation, thereby promoting the binding of circRNF20 to HuR and increasing the expression of CDCA4. The overexpression of circRNF20 or CDCA4 abolished the inhibitory effect of IGF2BP2 silencing on BC cell proliferation. In conclusion, the binding of IGF2BP2 to circRNF20 prevents its degradation, thus facilitating BC cell proliferation via the HuR/CDCA4 axis.

## 1 INTRODUCTION

Breast cancer (BC) is the most frequently diagnosed malignancy among women and is the second leading cause of cancer‐related mortality in the female population.^1^ BC encompasses a collection of biological subtypes exhibiting distinct molecular profiles, clinicopathological presentations, disease trajectories, and treatment outcomes.^2^ Epidemiological studies have correlated diverse spectra of risk factors for BC development or progression, including reproductive factors such as early menarche, nulliparity or late age at first birth, late menopause, family history, gene mutations, and unhealthy lifestyles.^3^ A deep understanding of BC nosogenesis has substantially improved early diagnosis and provided various treatment options, including surgery, endocrine therapy, chemotherapy, radiotherapy, targeted therapy, and immunotherapy.^4^ Nevertheless, the survival prognosis remains unsatisfactory, especially for triple‐negative BC (TNBC) and metastatic BC.^5^ Hence, determining more precise therapeutic targets is a pressing challenge with clinical implications.

Insulin‐like growth factor 2 mRNA‐binding protein 2 (IGF2BP2) is an evolutionarily conserved RNA‐binding protein in human eukaryotic cells that plays a vital role in multiple physiopathological processes, such as embryonic development, neurogenesis, energy metabolism, and carcinogenesis.^6^ IGF2BP2 promotes acute myeloid leukemia development and self‐renewal of leukemia stem/initiation cells by regulating critical targets (e.g., MYC, GPT2, and SLC1A5) in glutamine metabolism pathways.^7^ IGF2BP2 activates endothelial cells to promote angiogenesis and metastasis in lung adenocarcinoma.^8^ IGF2BP2 also aggravates colorectal cancer progression by increasing the stability of m6A‐modified CREB1 mRNA.^9^ In particular, IGF2BP2 has been deemed a BC susceptibility protein.^10^ IGF2BP2 accelerates BC cell proliferation and invasion by increasing the expression of the BC‐related genes IFIT2, CCL20, and CXCL10.^11^ IGF2BP2 also expedites the proliferation and G1/S phase transition of TNBC cells by directly affecting CDK6 m6A modification.^12^ IGF2BP2 stabilizes UBE2D1 mRNA in BC cells and then activates the TGF‐β/Smad2/3 signaling pathway to promote BC progression.^13^ These findings suggest the potential of IGF2BP2 as a BC therapeutic target and the necessity of further exploration of its mechanism in BC proliferation.

Circular RNAs (circRNAs) are newly identified endogenous noncoding RNA molecules with covalently closed loop structures generated from backsplicing of pre‐mRNAs.^14^ Aberrantly expressed circRNAs are closely linked with the initiation and progression of human malignancies, including BC.^15^ One such circRNA, circRNF20, serves as an oncogenic factor in non‐small cell lung carcinoma and retinoblastoma.^16^ CircRNF20 enhances BC carcinogenesis and Warburg effect via the miR‐487a/HIF‐1α/HK2 axis.^17^ A previous study provided evidence for the regulatory mechanism of IGF2BP2‐induced circRNA degradation in esophageal squamous cell carcinoma.^18^ However, whether IGF2BP2 affects the degradation of circRNF20 in BC cells remains unknown. The present study investigated the specific mechanism by which IGF2BP2 promotes BC cell proliferation and thereby provides a new theoretical basis for BC treatment.

## 2 MATERIALS AND METHODS

### 2.1 Cell culture

The human BC cell lines (MDA‐MB‐157, MDA‐MB‐468, MDA‐MB‐231, and MDA‐MB‐453) and the human normal breast epithelial cell line (MCF‐10A) were purchased from the American Type Culture Collection (ATCC, Manassas, VA, USA) and cultured in Dulbecco's modified Eagle's medium (DMEM) supplemented with 10% fetal bovine serum (Gibco, Grand Island, NY, USA) and 100 units/mL penicillin/streptomycin (Corning, NY, USA) at 37°C with 5% CO_2_.

### 2.2 Cell transfection

Negative controls (si‐NC) and small interfering RNAs (siRNAs) targeting IGF2BP2 (si‐IGF2BP2) or HuR (si‐HuR), control vectors and overexpression vectors targeting circRNF20 (circRNF20) or CDCA4 (CDCA4) were synthesized by the Gene‐Seed Biotechnology Co., Ltd. (Guangzhou, China). After reaching 80% confluence, the cells were used for transfection. Cell transfection was performed via the Lipofectamine 3000 reagent (Thermo Fisher Scientific, Waltham, MA, USA).

### 2.3 Cell counting kit‐8 (CCK‐8) assay

CCK‐8 assay was used to detect cell proliferation. Briefly, 1 × 10^3^ cells were seeded into 96‐well plates. At the designated times, the cells were incubated with 10 μL of CCK‐8 solution (Dojindo, Tokyo, Japan) per well. The absorbance value at 450 nm was measured with a microplate reader (Thermo Fisher Scientific).

### 2.4 Colony formation assay

Briefly, cells were seeded into six‐well plates (1 × 10^3^ cells/well) and maintained for 14 days. Then, the cells were washed three times with phosphate‐buffered saline (PBS), fixed in methanol for 30 min, and stained with crystal violet solution (Sigma‐Aldrich, St. Louis, MO, USA) for 30 min. Finally, the cell colonies were photographed and counted.

### 2.5 EdU staining

The cells (1 × 10^6^) were seeded into six‐well plates and cultured until the normal growth phase. After 48 h of transfection, the culture medium was replaced with fresh medium containing 50 μM EdU. After incubation for 2 h, the cells were washed twice with PBS (5 min/wash). Then, 100 μL of fixative (PBS containing 4% paraformaldehyde) was added to each well, and the mixture was incubated at room temperature for 30 min. The cells were neutralized with 2 mg/mL glycine for 5 min, washed once with PBS, and incubated at room temperature with 0.5% Triton X‐100 for 10 min. After washing with PBS, 100 μL of staining solution was added to each well, and the mixture was incubated at room temperature in the dark for 10 min. Finally, the cells were washed three times with PBS and photographed under a fluorescence microscope.

### 2.6 Bioinformatics analysis

The binding between IGF2BP2 and circRNF20, as well as that between circRNF20, HuR, and CDCA4, was predicted through the starBase database (https://rnasysu.com/encori/index.php).^19^ The RBPDB database (http://rbpdb.ccbr.utoronto.ca/)^20^ was used to predict the RNA‐binding proteins bound to circRNF20.

### 2.7 RNA immunoprecipitation (RIP)

RIP was performed via a RIP Assay Kit (Millipore, MA, USA). Approximately 2 × 10^7^ cells were harvested and lysed with RIP lysis buffer. The cell lysate was incubated with Pierce protein A/G magnetic beads (Thermo Fisher Scientific) bound to anti‐IGF2BP2 (ab117809, Abcam, Cambridge, MA, USA) or anti‐HuR (ab200342, Abcam) at 4°C overnight, with anti‐IgG (ab172730, Abcam) used as the negative control. Then, TRIzol reagent (Invitrogen, Carlsbad, CA, USA) was used to isolate coprecipitated RNA, and RNA abundance was evaluated by RT‐qPCR.

### 2.8 RNA pull‐down

The interaction between circRNF20 and HuR was validated by a biotin‐coupled circRNF20 probe and a control probe (GenePharma, Shanghai, China). Approximately 1 × 10^7^ cells were lysed and sonicated in a 4°C water bath for half an hour. A small portion (20 μL) of the lysate was allocated for RNA input, whereas the majority (80 μL) was reserved for protein input. The probe was placed into the lysis buffer and stirred at room temperature for 16–24 h. Then, 100 μL of streptavidin magnetic beads (MCE, Monmouth Junction, NJ, USA) were added to the lysis buffer and rotated at room temperature for 2–4 h. The magnetic beads were collected by a magnetic scaffold and washed five times with washing buffer (containing phenylmethylsulfonyl fluoride, protease inhibitor, and RNase inhibitor). The washed microspheres were resuspended in 1 mL of washing buffer, with 100 μL used for RNA purification and the remaining 900 μL used for protein purification. For RNA extraction, 100 μL of sample was mixed with 5 μL of proteinase K (Sangon Biotech, Shanghai, China) and RNA PK buffer, gently rotated at 50°C for 45 min, and then heated at 95°C for 10 min to disrupt formaldehyde cross‐linking. The RNA was subsequently purified using TRIzol reagent (Invitrogen), transcribed into cDNA, and stored at −80°C until use. The remaining 900 μL of sample was added to 300 μL of 4× loading buffer and incubated at 100°C for 10 min to extract protein. The supernatant containing the protein extract was separated using a magnetic scaffold, and the separated protein was subjected to Western blotting.

### 2.9 Colocalization of circRNF20 and HuR

The colocalization of circRNF20 and HuR in cells was evaluated using fluorescence staining. Approximately, 2000 cells were seeded into 48‐well plates. When the confluence reached 70%–90%, the cells were washed three times with PBS and fixed with 4% paraformaldehyde. The cells were subsequently permeabilized with 0.5% Triton X‐100 at 4°C for 10 min, prehybridized with prehybridization buffer at 37°C for 30 min, and then incubated overnight with a Cy3‐labeled circRNF20‐FISH probe (RiboBio, Guangzhou, China) in hybridization buffer at 37°C in the dark. Then, the cells were permeabilized again with 0.5% Triton X‐100 at 4°C for 10 min and incubated overnight with an anti‐HuR antibody (ab310967, Abcam) in the dark at 4°C. Afterwards, the cells were washed with PBS, and the nuclei were stained with 4′,6‐diamidino‐2‐phenylindole for 15 min. Finally, the cells were observed and imaged under a confocal fluorescence microscope (Carl Zeiss AG, Jenna, Germany).

### 2.10 Determination of mRNA stability

Briefly, 5 μg/mL actinomycin D (Sigma–Aldrich) was applied to the cells at 0, 4, 8, 12, and 24 h, respectively. Then, the cells were collected, and total RNA was extracted. RT–qPCR was performed to analyze the stability of circRNF20.

### 2.11 Nude mouse xenograft tumor experiment

The animal experiments were conducted under the guidance of the Animal Care and Use Committee of our hospital. All animal experimental procedures complied with the *Guidelines for the Care and Use of Laboratory Animals* of the National Institutes of Health.^21^ Male BALB/c nude mice (6 weeks old) were purchased from the Hunan SJA Experimental Animal Co., Ltd., and 2 × 10^7^/mL MDA‐MB‐231 cells infected with IGF2BP2 knockdown lentivirus (sh‐IGF2BP2) or negative control lentivirus (sh‐NC) (GenePharma, Shanghai, China) at a ratio of 20 were injected subcutaneously into the right flank. The tumor size was measured every 7 days. On the 28th day after xenografting, the mice were euthanized by intraperitoneal injection of 150 mg/kg pentobarbital sodium (volume = length × width^2^/2). The tumor tissues were excised and weighed for subsequent measurement.

### 2.12 Immunohistochemical staining

The tumor tissue samples were fixed, embedded in paraffin, dewaxed, rehydrated, and subjected to antigen extraction. The sections were incubated with Ki‐67 (ab15580, Abcam), stained with secondary antibody (ab205718, Abcam), visualized with DAB solution, counterstained with hematoxylin, and sealed. Finally, the glass slides were covered, sealed with neutral resin (Yeasen, Shanghai, China), and observed under a fluorescence microscope.

### 2.13 Real‐time reverse transcriptase–polymerase chain reaction (RT–qPCR)

Total RNA was isolated via the use of TRIzol reagent (Invitrogen). The concentrations of the RNA samples were measured using a NanoDrop spectrophotometer (NanoDrop Technologies, Thermo Fisher Scientific), and the isolated RNA was reverse transcribed into cDNA using oligonucleotide (dT) primers and reverse transcriptase (Promega, Madison, WI, USA). Real‐time PCR was performed via a SYBR Premix Ex Taq Assay Kit (Takara, Otsu, Japan) on an ABI 7500 instrument (Applied Biosystems; Thermo Fisher Scientific). The relative expression of genes was calculated using the 2^−ΔΔCt^ method^22^ with GAPDH as the internal reference. The primers used are shown in Table 1.

**TABLE 1:** PCR primer sequences.

Name	Sequence (5′–3′)
IGF2BP2	F: AGAATGTGGAACAAGTCAACACA
	R: TTCTCAAACTGATGCCCGCT
circRNF20	F: CAGGGCTTGGGAGACCTACT
	R: CCATCACGAACACTTGCCTCT
HuR	F: CAGATGTTTGGGCCGTTTGG
	R: TGGTCACAAAGCCAAACCCT
CDCA4	F: GGATGGCCCTCGAGAAAACA
	R: TGTCCACGTCTGAGAACAGC
GAPDH	F: GATGCTGGCGCTGAGTACG
	R: GCTAAGCAGTTGGTGGTGC

*Note*: CDCA4, cell division cycle‐associated protein 4; circRNF20, circRNA ring finger protein 20; GAPDH, glyceraldehyde‐3‐phosphate dehydrogenase; HuR, Hu‐antigen R; IGF2BP2, insulin‐like growth factor 2 mRNA binding protein 2.

### 2.14 Western blotting

Protein was extracted in radioimmunoprecipitation lysis buffer (Beyotime, Shanghai, China). After protein quantification by a bicinchoninic acid assay (Thermo Fisher Scientific), 20 μg of protein sample was separated by 10% SDS‐PAGE and transferred onto polyvinylidene fluoride membranes (Immobilon‐P; Merck Millipore, Darmstadt, Germany). The membranes were blocked with 5% skim milk and incubated overnight with primary antibodies against IGF2BP2 (ab117809, 1:2000, Abcam), HuR (ab200342, 1:1000, Abcam), CDCA4 (11625‐1‐AP, 1:1000, Proteintech), and β‐actin (ab5694, 1:1000, Abcam) at 4°C, followed by incubation with a secondary antibody (ab205718, 1:2000, Abcam) at 26°C for 1 h. Pierce ECL Western blotting Substrate (Thermo Fisher Scientific) was used to visualize the protein bands. The relative expression was calculated using ImageJ software (version 1.50), with β‐actin used as an endogenous control.

### 2.15 Statistical analysis

Data analysis and map plotting were performed using SPSS 21.0 (IBM Corp., Armonk, NY, USA) and GraphPad Prism 8.0 (GraphPad Software Inc., San Diego, CA, USA). The data were examined for a normal distribution and homogeneity of variance. The t‐ test was used for comparisons between two groups, and one‐way or two‐way analysis of variance (ANOVA) was used for comparisons among multiple groups, followed by Tukey's multiple comparison test. A value of *p* < 0.05 indicated a significant difference.

## 3 RESULTS

### 3.1 IGF2BP2 is highly expressed in BC and facilitates BC cell proliferation

**FIGURE 1:**
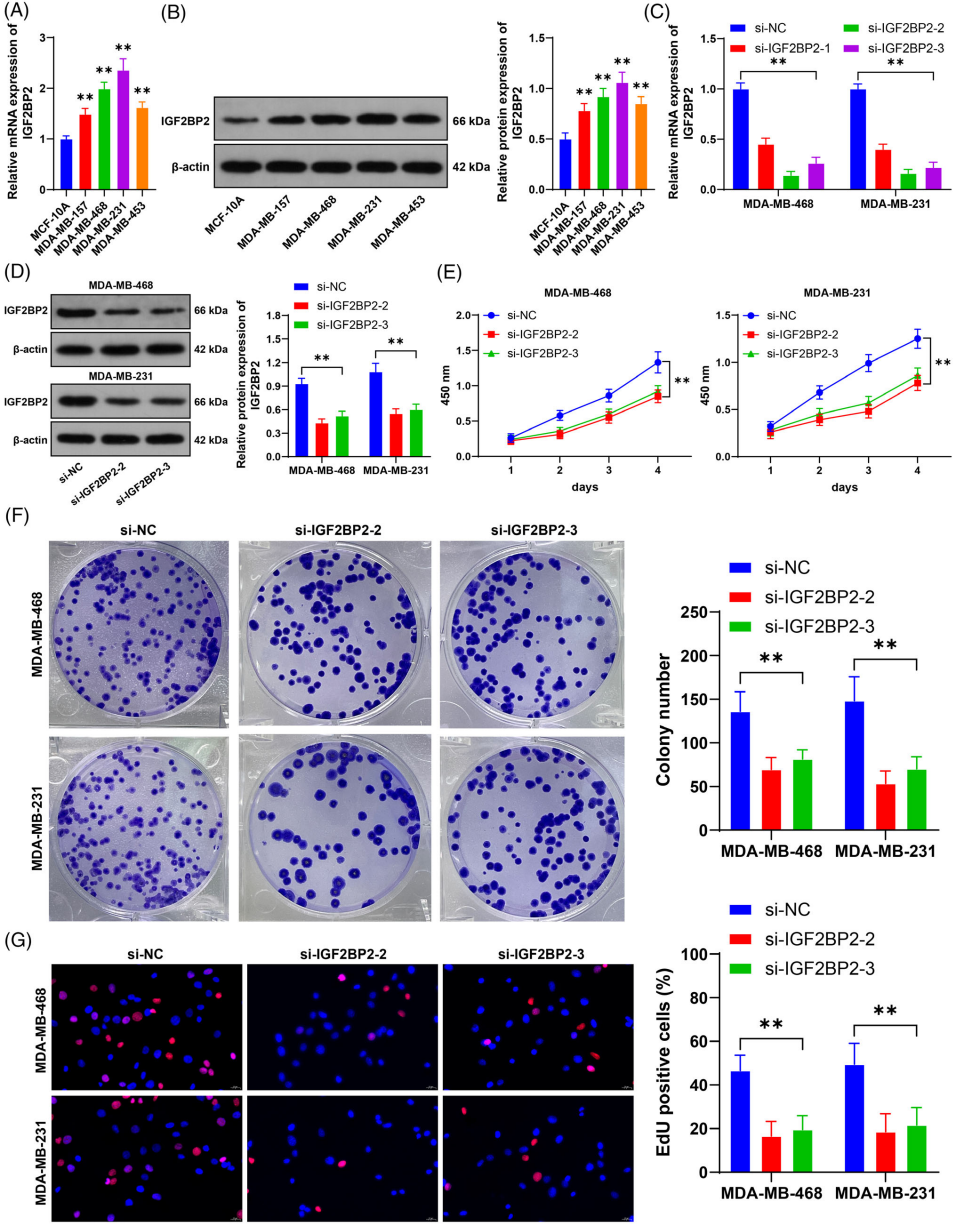
IGF2BP2 is highly expressed in BC and promotes BC cell proliferation. (A,B) IGF2BP2 expression in cell lines was detected by RT–qPCR and Western blotting; si‐IGF2BP2 was transfected into cells, with si‐NC used as a negative control. (C,D) IGF2BP2 expression in cells was detected by RT–qPCR and Western blotting. (E) Cell proliferation was evaluated by a CCK‐8 assay. (F) The number of cell colonies was detected via colony formation assay. (G) The number of EdU‐positive cells was detected by EdU staining. The cell experiments were independently repeated three times. The data are expressed as the means ± standard deviations. (A,B) **p < 0.01 versus MCF‐10A, others: **p < 0.01. The data in panels (A) and (B) were analyzed by one‐way ANOVA, and the data in panels C–G were analyzed by two‐way ANOVA, followed by Tukey's multiple comparisons test.

Compared with that in MCF‐10A cells, IGF2BP2 expression in BC cell lines was notably increased (*p* < 0.05, Figure 1A,B). We successfully reduced IGF2BP2 expression in MDA‐MB‐468 and MDA‐MB‐231 cells with relatively high IGF2BP2 expression by transfecting them with an IGF2BP2 siRNA (si‐IGF2BP2) (*p* < 0.05, Figure 1C,D), which included two siRNAs with favorable transfection efficiency for functional validation. The results revealed that silencing IGF2BP2 significantly decreased cell proliferation (*p* < 0.05, Figure 1E). Moreover, silencing IGF2BP2 reduced the number of cell colonies and the number of EdU‐positive cells (*p* < 0.05, Figure 1F,G). Taken together, these results indicate that IGF2BP2 is highly expressed in BC and promotes BC cell proliferation.

### 3.2 Binding of IGF2BP2 to circRNF20 prevents its degradation

**FIGURE 2:**
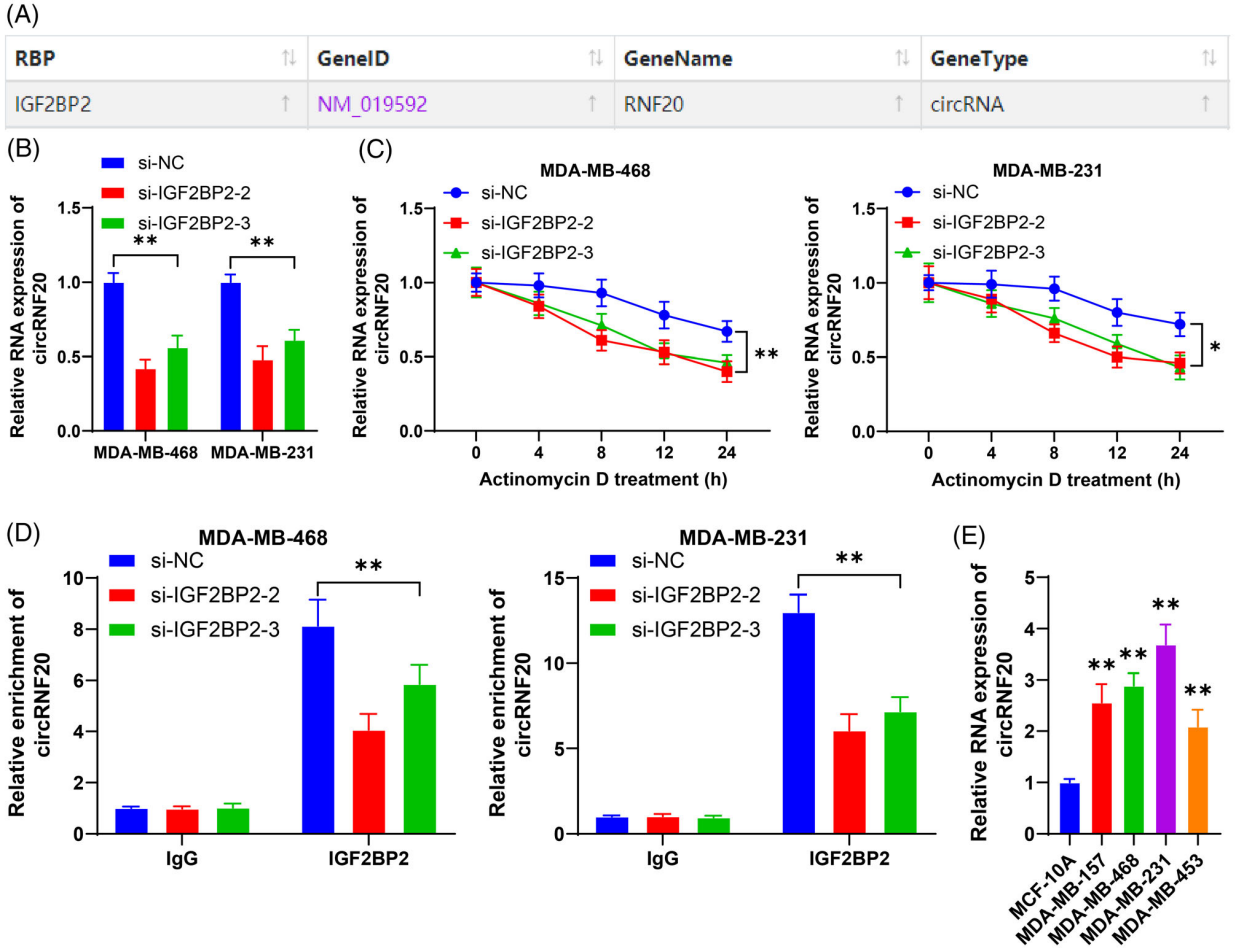
The binding of IGF2BP2 to circRNF20 prevents its degradation. (A) circRNA downsream of IGF2BP2 was predicted through the Starbase database. (B) circRNF20 expression in cells was detected by RT–qPCR. (C) After treatment with actinomycin D, circRNF20 expression in cells was detected via RT–qPCR. (D) The binding between IGF2BP2 and circRNF20 was detected by RIP. (E) circRNF20 expression in cells was detected by RT–qPCR. The cell experiments were independently repeated three times. The data are expressed as the means ± standard deviations. (E) **p < 0.01 versus MCF‐10A, others: *p < 0.05, **p < 0.01. The data in panel (E) were analyzed by one‐way ANOVA, and the data in panels B‐D were analyzed by two‐way ANOVA, followed by Tukey's multiple comparisons test.

The binding of IGF2BP2 to circRUNX1 prevents its degradation.^18^ CircRNAs downstream of IGF2BP2 were predicted through the starBase database (Figure 2A), among which circRNF20 is upregulated in BC tissues and cells.^17^ However, whether IGF2BP2 affects the degradation of circRNF20 is still unclear. As expected, the RT–qPCR results demonstrated that the knockdown of IGF2BP2 reduced circRNF20 expression (*p* < 0.05, Figure 2B). To determine the inhibitory effect of IGF2BP2 on circRNF20 degradation, we treated cells with actinomycin D at specific time points. Notably, silencing IGF2BP2 significantly diminished the half‐life of circRNF20 (*p* < 0.05, Figure 2C). Subsequently, RIP confirmed the direct interaction between IGF2BP2 and circRNF20 in BC cells (*p* < 0.05, Figure 2D). Compared with that in MCF‐10A cells, circRNF20 expression was significantly elevated in BC cell lines (*p* < 0.05, Figure 2E). These findings indicate that IGF2BP2 serves as an upstream target of circRNF20 and promotes circRNF20 expression by increasing its RNA stability.

### 3.3 Overexpression of circRNF20 alleviates the inhibitory effect of IGF2BP2 silencing on BC cell proliferation

**FIGURE 3:**
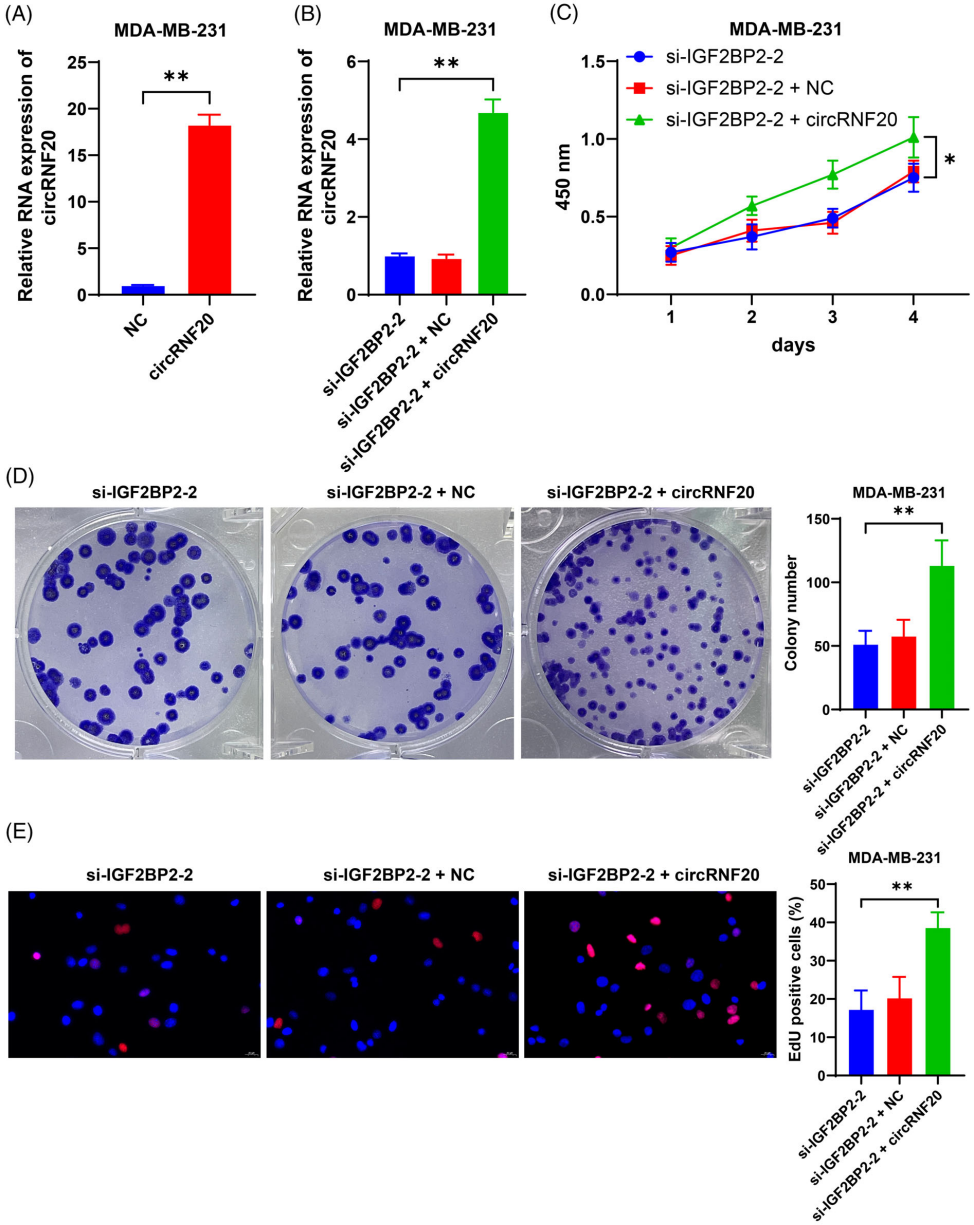
Overexpression of circRNF20 alleviates the inhibitory effect of IGF2BP2 silencing on BC cell proliferation. An overexpression vector targeting circRNF20 was transfected into cells, with NC as a negative control. (A,B) circRNF20 expression in cells was detected by RT–qPCR. (C) Cell proliferation was evaluated by CCK‐8 assay. (D) The number of cell colonies was detected via colony formation assay. (E) The number of EdU‐positive cells was detected by EdU staining. The cell experiments were independently repeated three times. The data are expressed as the means ± standard deviations. *p < 0.05, **p < 0.01. The data in panel (A) were analyzed by a t test. The data in panels (B), (D), and (E) were analyzed by one‐way ANOVA, and the data in panel (C) were analyzed by two‐way ANOVA, followed by Tukey's multiple comparisons test.

Furthermore, we upregulated circRNF20 expression in MDA‐MB‐231 cells (*p* < 0.05, Figure 3A) and performed a combined experiment with si‐IGF2BP2‐2 (*p* < 0.05, Figure 3B). As indicated by the CCK‐8 results, circRNF20 overexpression significantly increased cell proliferation (*p* < 0.05, Figure 3C). Compared with si‐IGF2BP2‐2 alone, the combined treatment resulted in a significant increase in the number of cell colonies and EdU‐positive cells (*p* < 0.05, Figure 3D,E). Briefly, overexpression of circRNF20 alleviated the inhibitory effect of IGF2BP2 silencing on BC cell proliferation.

### 3.4 The binding of circRNF20 and HuR promotes the expression of CDCA4

**FIGURE 4:**
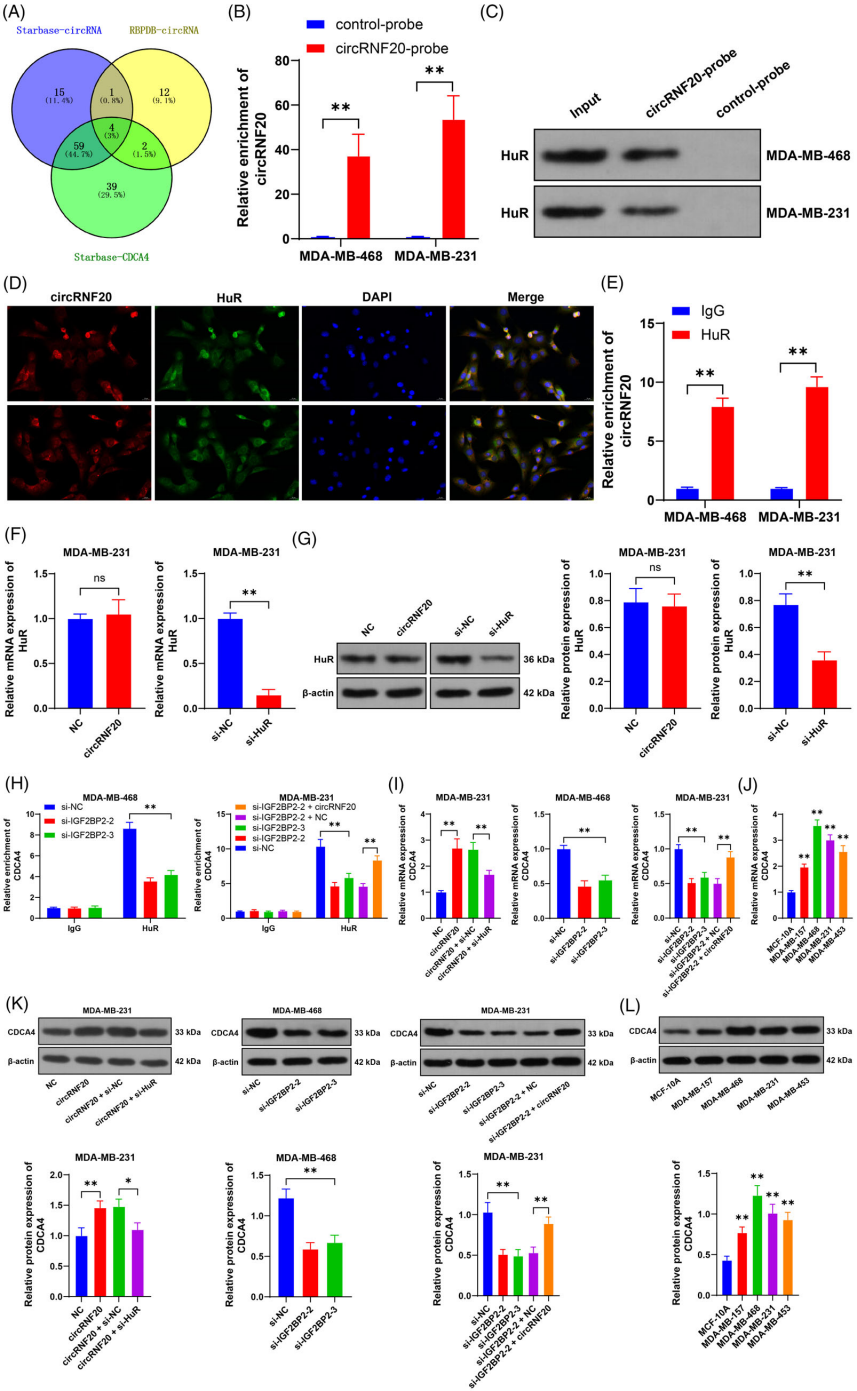
The binding of circRNF20 and HuR promotes the expression of CDCA4. (A) The RNA‐binding proteins bound to circRNF20 and CDCA4 were predicted through the starBase and RBPDB databases. (B,C) The binding between circRNF20 and HuR was analyzed via RNA pull‐down assay. (D) The cell localization of circRNF20 and HuR was detected by FISH‐IF. (E) The binding between HuR and circRNF20 was detected by RIP. (F,G) HuR expression in cells was detected by RT–qPCR and Western blotting. (H) The binding between HuR and CDCA4 was detected by RIP. (I–L) CDCA4 expression in cells was detected by RT–qPCR and Western blotting. The cell experiments were independently repeated three times. The data are expressed as the means ± standard deviations. (J,L) **p < .01 versus MCF‐10A, others: *p < 0.05, **p < 0.01. The data in panels (F) and (G) were analyzed by a *t* test. The data in panels (I–L) were analyzed by one‐way ANOVA, and the data in panels (B), (E), and (H) were analyzed by two‐way ANOVA, followed by Tukey's multiple comparisons test.

The binding of circRNAs to RNA‐binding proteins facilitates the expression of downstream genes.^23^ CDCA4 is abundantly expressed in BC.^24^, ^25^ We predicted that RNA‐binding proteins bind to circRNF20 and CDCA4 through the databases and found that HuR was the most common RNA‐binding protein at the intersection (Figure 4A). In the RNA pull‐down assay, circRNF20 was specifically enriched in the circRNF20 probe group (*p* < 0.05, Figure 4B). Western blot results revealed that circRNF20 could specifically bind to HuR (Figure 4C). FISH‐IF detection revealed that circRNF20 and HuR were concentrated mainly in the cytoplasm of BC cells (Figure 4D). The RIP results indicated that circRNF20 interacted with HuR (*p* < 0.05, Figure 4E). In addition, circRNF20 overexpression did not affect the protein or RNA levels of HuR (*p* < 0.05, Figure 4F,G). The RIP results revealed that HuR could bind to CDCA4, while silencing IGF2BP2 reduced the binding between HuR and CDCA4, and overexpression of circRNF20 increased the binding between HuR and CDCA4 (*p* < 0.05, Figure 4H). The overexpression of circRNF20 increased the expression of CDCA4, whereas the silencing of HuR decreased the increase in CDCA4 expression (*p* < 0.05, Figure 4F,G,I,J). In addition, CDCA4 expression was elevated in BC cells but decreased in cells with silenced IGF2BP2 and increased in cells overexpressing circRNF20 (*p* < 0.05, Figure 4I,J). These results reveal that the binding of circRNF20 to HuR promotes the expression of CDCA4.

### 3.5 Overexpression of CDCA4 alleviates the inhibitory effect of IGF2BP2 silencing on BC cell proliferation

**FIGURE 5:**
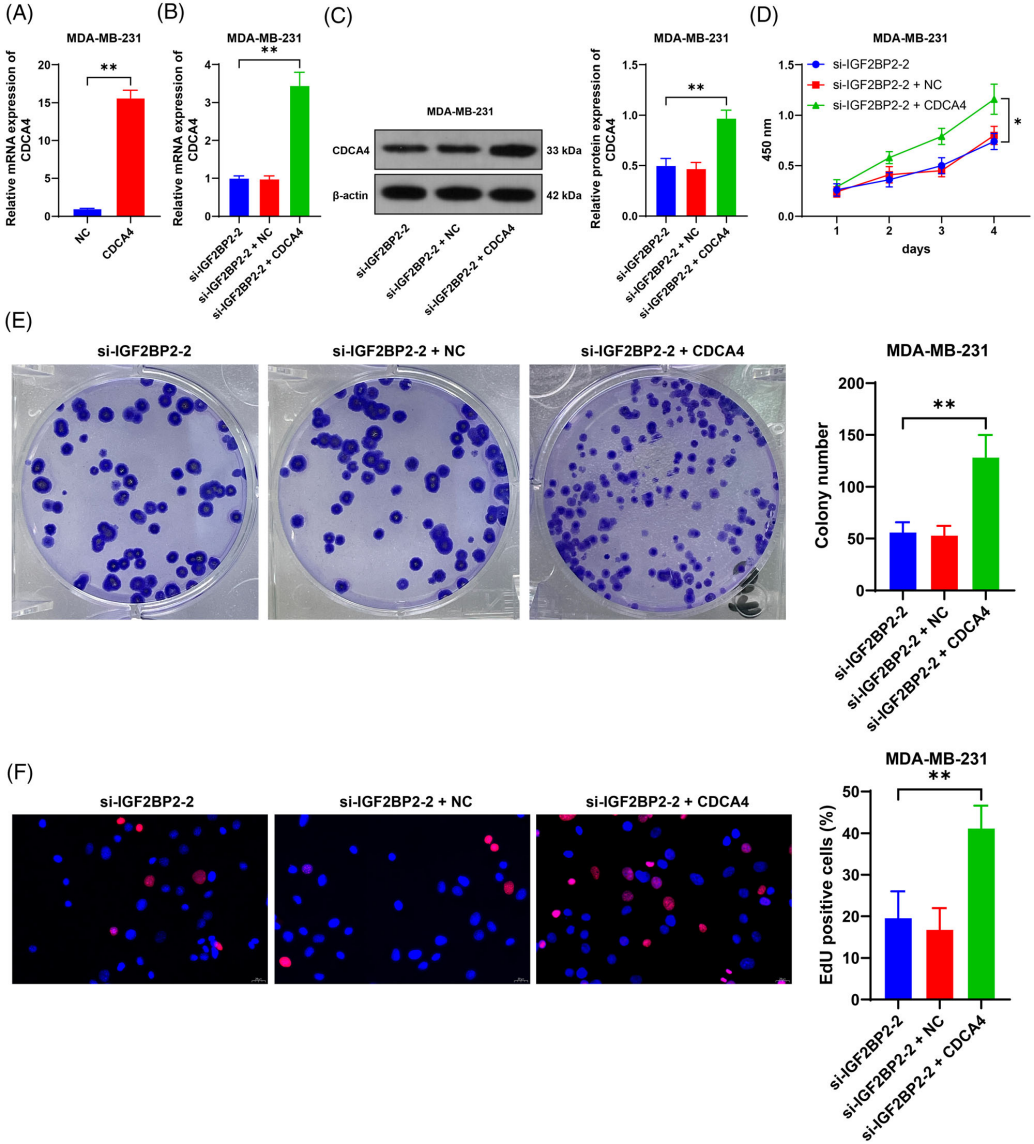
Overexpression of CDCA4 alleviates the inhibitory effect of IGF2BP2 silencing on BC cell proliferation. An overexpression vector targeting CDCA4 (CDCA4) was transfected into cells, with NC as a negative control. (A–C) CDCA4 expression in cells was detected by RT–qPCR and Western blotting. (D) Cell proliferation was evaluated by CCK‐8 assay. (E) The number of cell colonies was detected via colony formation assay. (F) The number of EdU‐positive cells was detected by EdU staining. The cell experiments were independently repeated three times. The data are expressed as the means ± standard deviations. *p < 0.05, **p < 0.01. The data in panel (A) were analyzed by a *t* test. The data in panels (B), (C), (E), and (F) were analyzed by one‐way ANOVA, and the data in panel (D) were analyzed by two‐way ANOVA, followed by Tukey's multiple comparisons test.

Finally, we upregulated CDCA4 expression in MDA‐MB‐231 cells (*p* < 0.05, Figure 5A), followed by combined treatment with si‐IGF2BP2‐2 (*p* < 0.05, Figure 5B,C). The overexpression of CDCA4 led to a significant increase in proliferation, colony formation, and the number of EdU‐positive cells (*p* < 0.05, Figure 5D,F). These results suggest that the overexpression of CDCA4 alleviates the inhibitory effect of IGF2BP2 silencing on BC cell proliferation.

### 3.6 Low expression of IGF2BP2 inhibits BC proliferation in vivo by suppressing the circRNF20/CDCA4 axis

**FIGURE 6:**
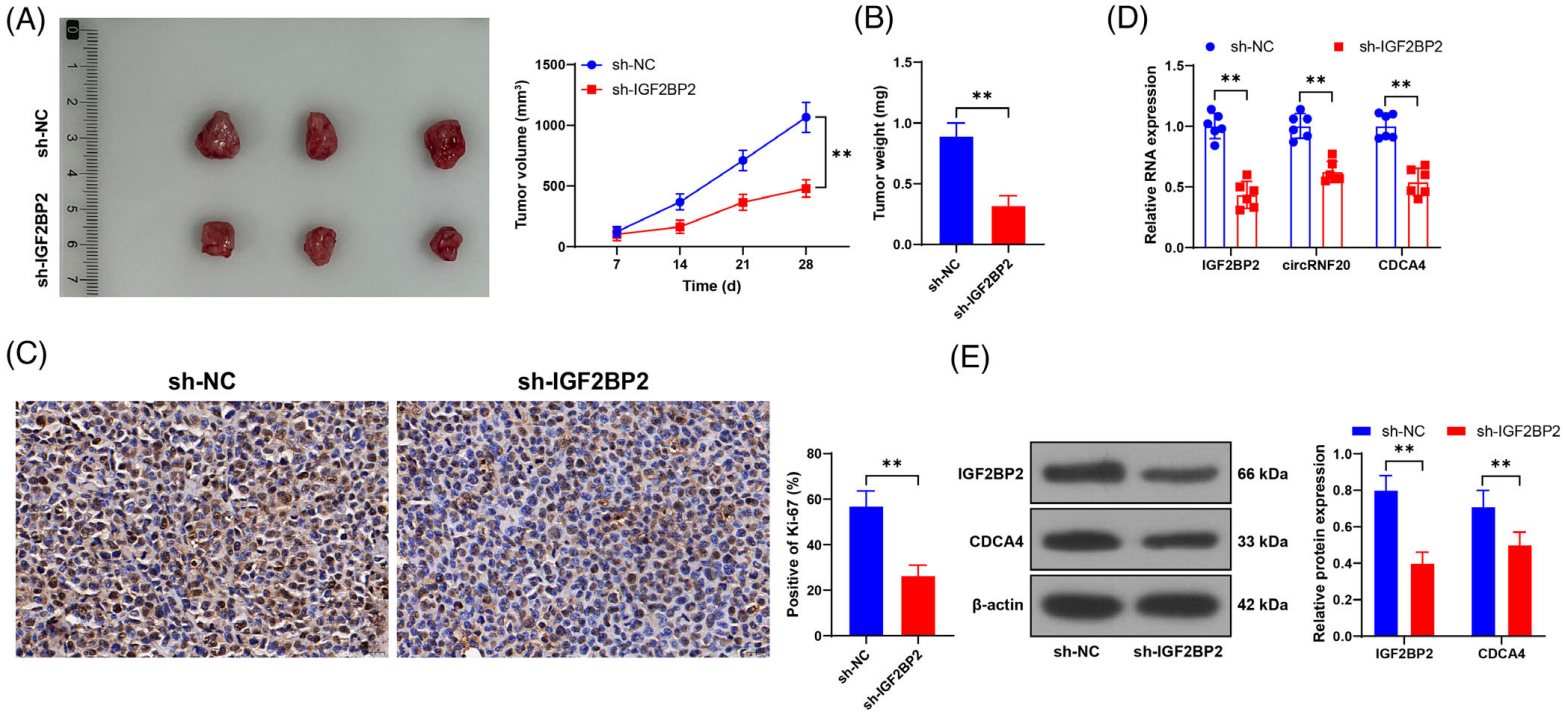
Low expression of IGF2BP2 inhibits BC proliferation in vivo by suppressing the circRNF20/CDCA4 axis. A nude mouse xenograft tumor model was constructed by injecting MDA‐MB‐231 cells infected with IGF2BP2 shRNA lentivirus (sh‐IGF2BP2), with MDA‐MB‐231 cells infected with sh‐NC as a negative control. (A) The tumor volume was measured every 7 days, and representative images were taken after the nude mice were euthanized on the 28th day. (B) Tumor weight. (C) Immunohistochemical detection of the Ki‐67 positivity rate. (D) The expression levels of IGF2BP2, circRNF20, and CDCA4 were detected via RT–qPCR. (E) The expression levels of IGF2BP2 and CDCA4 were detected by Western blotting. *N* = 6. The cell experiments were independently repeated three times. The data are expressed as the means ± standard deviations. **p < 0.01. The data in panels (B) and (C) were analyzed by *t* tests. The data in panels (A), (D), and (E) were analyzed by two‐way ANOVA, followed by Tukey's multiple comparisons test.

Finally, we constructed a nude mouse xenograft tumor model using MDA‐MB‐231 cells infected with sh‐IGF2BP2 and reported that low expression of IGF2BP2 resulted in decreases in tumor volume and weight (*p* < 0.05, Figure 6A,B). Compared with those in the sh‐NC group, the percentage of Ki‐67‐positive tumor tissues in the sh‐IGF2BP2 group was lower (*p* < 0.05, Figure 6C), and the expression levels of IGF2BP2, circRNF20, and CDCA4 were lower (*p* < 0.05, Figure 6D,E). In summary, low expression of IGF2BP2 inhibits BC proliferation in vivo by suppressing the circRNF20/CDCA4 axis.

## 4 DISCUSSION

**FIGURE 7:**
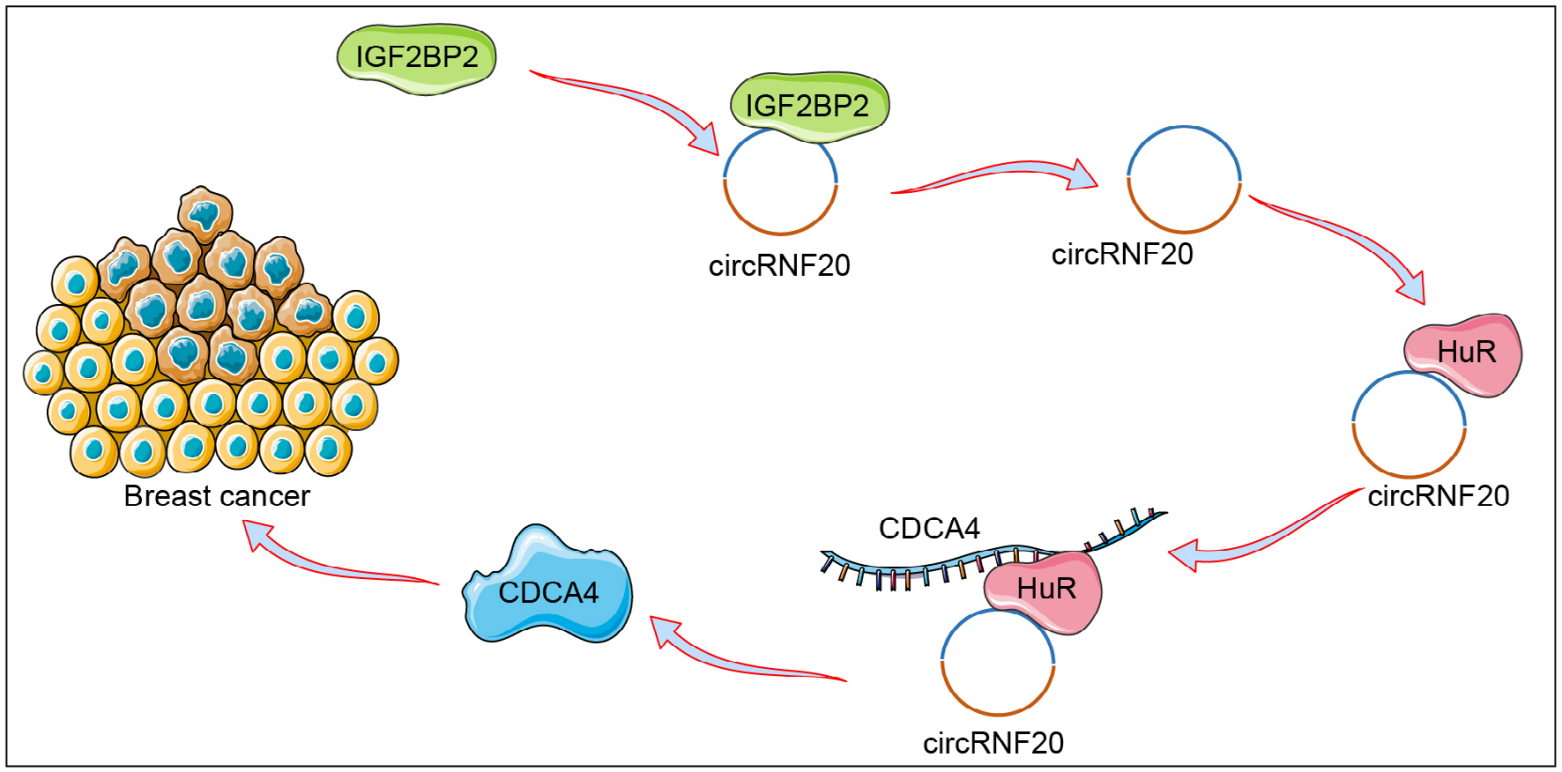
IGF2BP2 promotes BC cell proliferation via the circRNF20‐CDCA4 axis. The binding of IGF2BP2 to circRNF20 promotes the expression of circRNF20, which in turn increases the expression of CDCA4 by binding to HuR and promoting the proliferation of BC cells.

BC remains one of the primary causes of cancer‐related deaths among postmenopausal women worldwide, particularly in underdeveloped countries.^5^ A body of evidence exists supporting IGF2BP2 as a contributor to BC carcinogenesis and subsequent progression.^26^, ^27^, ^28^ This study revealed that IGF2BP2‐induced circRNF20 facilitates BC cell proliferation via the HuR/CDCA4 axis (Figure 7).

IGF2BP2 is highly expressed in BC, and knockdown of IGF2BP2 represses BC cell proliferation.^10^ IGF2BP2‐mediated m6A modification increases UBE2D1 mRNA stability, thus facilitating BC progression via the TGF‐β/Smad2/3 pathway.^13^ IGF2BP2 promotes cell cycle progression in TNBC by recruiting EIF4A1 to facilitate m6A‐modified CDK6 translation.^12^ Consistently, our results demonstrated that IGF2BP2 was highly expressed in BC and promoted BC cell proliferation. Emerging evidence reveals that IGF2BP2 participates in the development and progression of cancers by communicating with different RNAs, such as (miRNAs), lncRNAs, and circRNAs.^29^ CircRNA microarray analysis revealed that circRNF20 is an elevated RNA transcript in BC.^17^ We also noted that circRNF20 expression was dramatically elevated in BC cells and that knockdown of IGF2BP2 reduced circRNF20 expression. A previous study revealed that IGF2BP2 functions as an upstream target of circRUNX1 to suppress its degradation.^18^ Accordingly, we speculated that IGF2BP2 affects the degradation of circRNF20 in BC. To determine the inhibitory effect of IGF2BP2 on circRNF20 degradation, we treated cells with actinomycin D at specific time points. Notably, silencing IGF2BP2 significantly diminished the half‐life of circRNF20. Subsequently, RIP confirmed the direct interaction between IGF2BP2 and circRNF20 in BC cells. Functional rescue experiments demonstrated that circRNF20 overexpression abolished the inhibitory effect of IGF2BP2 silencing on BC cell proliferation. Similarly, previous results indicate that circRNF20 overexpression expedites proliferation but decreases apoptosis in BC cells.^17^

Therefore, we investigated the downstream genes regulated by circRNF20 in BC cell proliferation. CDCA4 belongs to the family of selected INK4A (SEI) genes and critically participates in cell cycle progression by coexpressing with cell cycle genes such as CDC2, CDC7, and cyclins.^30^ Furthermore, a plethora of studies have revealed the intrinsic association between CDCA4 overexpression and tumorigenesis.^31^ CDCA4 interacts with IGF2BP1 to regulate lung adenocarcinoma proliferation via the PI3K/AKT pathway.^32^ Increased CDCA4 expression is positively correlated with increased T, N, and pathological stages and worse primary therapy outcomes in patients with lung adenocarcinoma.^33^ CDCA4 is highly expressed in BC, and CDCA4 silencing dramatically enhances the apoptosis and slows the proliferation of BC cells in vitro.^24^, ^25^, ^34^ By controlling the cell cycle, adriamycin resistance in BC cells is partially altered via the regulation of CDCA4, which may provide some novel insights for BC chemotherapy.^35^ We also found that CDCA4 expression was elevated in BC cells but decreased after the silencing of IGF2BP2, and the overexpression of circRNF20 increased CDCA4 expression. The overexpression of CDCA4 counteracted the inhibitory effect of IGF2BP2 silencing on BC cell proliferation. Furthermore, we predicted that RNA‐binding proteins bind to circRNF20 and CDCA4 through a database, among which HuR is the most commonly used RNA‐binding protein at the intersection. HuR is an extensively expressed RNA‐binding posttranscriptional regulator^36^ that is modulated by various proteins, microRNAs, and chemical compounds, thereby impacting the mRNA stability and translation of genes involved in BC carcinogenesis, metastasis, and treatment.^37^, ^38^, ^39^ In addition, clinical studies have revealed that HuR expression is elevated at all stages of BC and is correlated with aggressive forms of BC.^36^ Our RIP results verified the interactions between circRNF20‐HuR and HuR‐CDCA4. The silencing of IGF2BP2 decreased the binding of HuR to CDCA4, whereas the overexpression of circRNF20 led to the opposite trend. In addition, the overexpression of circRNF20 increased the expression of CDCA4, whereas the silencing of HuR resulted in the opposite results. These results indicate that the binding of circRNF20 to HuR enhances the expression of CDCA4.

In summary, the binding of IGF2BP2 to circRNF20 prevents its degradation, thus facilitating BC cell proliferation via the HuR/CDCA4 axis. Considering that the present study is a preliminary exploration of BC cell proliferation, the mechanism revealed is slightly simpler and has only been validated at the cellular level. However, whether IGF2BP2 regulates ferroptosis, apoptosis, autophagy, and other cellular processes in BC cells remains unclear. circRNF20 can function through a competitive endogenous RNA mechanism. In addition, we have not yet combined the role of IGF2BP2 with clinical practice. In the future, we will further explore the downstream mechanisms of circRNF20 and include clinical samples if conditions permit to provide new theoretical knowledge for the treatment of BC.

## CONFLICT OF INTEREST STATEMENT

The authors declare no conflict of interest.

## Data Availability

The data that support the findings of this study are available from the corresponding author upon reasonable request.
